# Coenzyme Q10 trapping in mitochondrial complex I underlies Leber’s hereditary optic neuropathy

**DOI:** 10.1073/pnas.2304884120

**Published:** 2023-09-21

**Authors:** Jack T. Fuller, Steven Barnes, Lorenzo A. Sadun, Pujan Ajmera, Anastassia N. Alexandrova, Alfredo A. Sadun

**Affiliations:** ^a^Department of Chemistry and Biochemistry, University of California, Los Angeles, CA 90095; ^b^Department of Ophthalmology, David Geffen School of Medicine, University of California, Los Angeles, CA 90095; ^c^Doheny Eye Institute, Pasadena, CA 91103; ^d^Department of Neurobiology, David Geffen School of Medicine, University of California, Los Angeles, CA 90095; ^e^Department of Mathematics, University of Texas at Austin, Austin, TX 78712

**Keywords:** Coenzyme Q10, mitochondria, molecular dynamics simulation, quantum electron tunneling, blinding genetic disease

## Abstract

How does a single amino acid mutation occurring in the blinding disease, Leber’s hereditary optic neuropathy (LHON), impair electron shuttling in mitochondria? We investigated changes induced by the m.3460 G>A mutation in mitochondrial protein ND1 using the tools of Molecular Dynamics and Free Energy Perturbation simulations, with the goal of determining the mechanism by which this mutation affects mitochondrial function. A recent analysis suggested that the mutation’s replacement of alanine A52 with a threonine perturbs the stability of a region where binding of the electron shuttling protein, Coenzyme Q10, occurs. We found two functionally opposing changes involving the role of Coenzyme Q10. The first showed that quantum electron transfer from the terminal Fe/S complex, N2, to the Coenzyme Q10 headgroup, docked in its binding pocket, is enhanced. However, this positive adjustment is overshadowed by our finding that the mobility of Coenzyme Q10 in its oxidized and reduced states, entering and exiting its binding pocket, is disrupted by the mutation in a manner that leads to conditions promoting the generation of reactive oxygen species. An increase in reactive oxygen species caused by the LHON mutation has been proposed to be responsible for this optic neuropathy.

## Significance

In the genetic disease Leber’s hereditary optic neuropathy, inheritance of a specific, single mitochondrial DNA mutation can produce the sudden onset of permanent blindness in first one and then the other eye usually in young adulthood. The mutation affects proteins in mitochondrial complex I that produce energy for retinal neuron activity, but the mechanism by which the mutation causes mitochondrial dysfunction is unclear. Computational biochemistry tools were used to compare molecular interactions in the mutated protein and showed that electron transfer to Coenzyme Q10 is massively slowed, creating conditions favorable for the production of cell-damaging reactive oxygen species, providing an explanation of how the mutation disrupts mitochondrial function, initiating a cascade that may lead to blindness.

Leber's hereditary optic neuropathy (LHON) is a maternally inherited genetic disorder that causes asynchronous, severe bilateral loss of vision especially in young adult males. Symptoms include rapid loss of visual acuity, dyschromatopsia, dense central scotoma, and optic atrophy (1, 2). This is known to be caused by one of several different mutations in the electron transport chain of mitochondria (3, 4). The mitochondrial DNA (mtDNA) mutation is necessary but not sufficient to cause vision loss through this optic neuropathy. Many carriers remain asymptomatic, with males more likely than females to lose sight, typically near the age of 20.

Aerobic ATP synthesis by mitochondria utilizes the energy from electron transfer including those from reduced nicotinamide adenine dinucleotide (NADH) to Coenzyme Q10 (CoQ10) to produce a proton gradient across the mitochondrial inner membrane. CoQ10 is extremely lipid soluble and relies on a binding channel within complex 1 to enable its redox-active quinone headgroup to be stable at a point about 20 Å above the membrane surface, precisely positioned to receive electrons from the terminal Fe-S complex, N2, in complex 1. CoQ10 reduction occurs where the electron and proton transfer assemblies of complex 1 are most critical. This juxtaposition creates a crux for disease-causing mutations to interfere with electron transfer in a manner that promotes electron leakage, creating superoxide and other reactive oxygen species (ROS) (4).

The LHON mutation known to produce the largest biochemical impairments evidenced in human patient cybrid cell models (2, 5) is the one at mtDNA nucleotide position m.3460 of the ND1 protein of complex I (6). The G>A mutation leads to the substitution of alanine with threonine at position 52. This results in a relatively modest loss of energy production (ATP), but a more severe increase in ROS production which appears to be the basis of injury to retinal ganglion cells (RGCs) that leads to blindness (7). ROS production occurring at complex I is largely due to impaired transfer of electrons to CoQ10. A mouse model of LHON caused by this mutation has demonstrated a decreased complex I activity, increased ROS production, and little or no decrease of ATP production (8), very similar findings to those in patient-derived cybrid cells (5).

This tight-fitting binding channel in ND1 of complex I accommodates most of the ∼50-Å-long CoQ10 molecular chain, supporting rapid ingress in its oxidized state (as a quinone) and its regress when reduced, bearing 1 or 2 electrons and protons (as a quinol) (9). The channel appears to optimize the binding affinity of oxidized CoQ10 while providing an environment in which reduced CoQ10 dissociation is not rate-determining. Reduction of bound CoQ10 is the rate-limiting step for catalysis by complex 1 (9), and in this report, we show how the single-site, m.3460G>A mutation form of LHON naturally impairs egress, indicating that this aspect of complex I efficiency is the cause of energetic dysfunction in mutation carriers.

In mitochondrial complex I (NADH ubiquinone oxidoreductase), two electrons are transferred from NADH to a lipid-soluble carrier, CoQ10. After leaving its binding channel, the reduced product, CoQ10H_2_, (ubiquinol), freely diffuses within the membrane, delivering the electrons to complex III where further contributions to the proton gradient across the inner mitochondrial membrane are made. By what mechanism(s) does the mutation perturb the function of ND1? We show here that the m.3460G>A mutation, a known cause of LHON, produces, among other actions, a change in ND1 that projects the OH and CH_3_ side groups of the new threonine directly into the channel, dramatically slowing the rate at which CoQ10 can pass through the channel, thereby impairing the mechanism mediating electron transfer from the terminal Fe/S cluster, N2.

## Results and Discussion

**Fig. 1.**
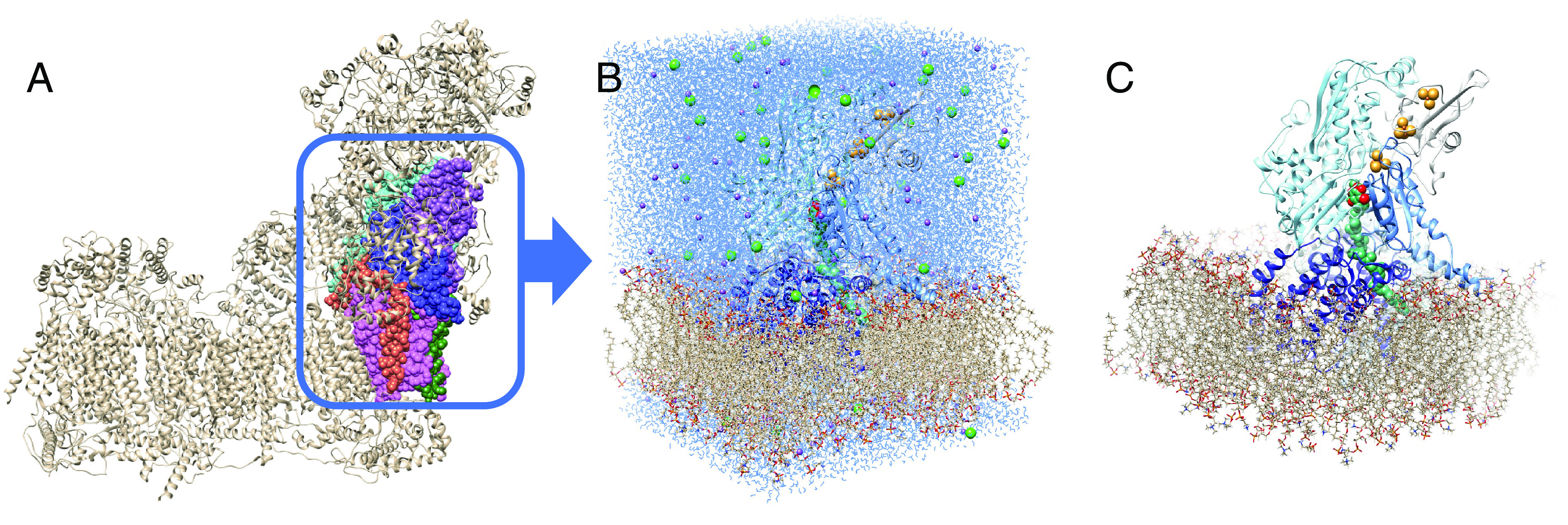
(*A*) The full complex I taken from the cryo-EM structure 5XTD, with the chains used in the simulations shown in color. (*B*) The periodic image of the fully solvated structure, including the colored part of the images in *A*, the membrane, and the Fe/S clusters. (*C*) Image with the solvent and electrolyte ions removed for clarity.

We performed molecular dynamics simulations of the CoQ10-ND1 complex for the native protein and the mutant at 310 K and 1.01325 bar using stochastic velocity rescaling (10) and Nosé–Hoover Langevin piston pressure control (11, 12) (see *Methods* for details). We used a truncated model of human complex I taken from the cryo-EM structure 5XTD (13). We included only chain B residues 58–210 (NDUFS8), chain C residues 58–213 (NDUFS7), chain Q residues 80–463 (NDUFS2), chain S residues 1–30 (NDUFA1), chain j residues 1–50 (ND3), and chain s residues 1–318 (ND1). We also included the last three 4Fe-4S clusters (N6a, N6b, and N2) (Fig. 1*A*). CoQ10 in its oxidized or reduced (quinone or quinol) forms was docked manually in the binding channel and subjected to equilibration. CHARMM-GUI (14–17) was used to build a membrane around the truncated model composed of 130 palmitoyl-oleyl-phosphatidylcholine molecules, 91 palmitoyl-oleyl-phosphatidylethanolamine molecules, and 39 tetra-hexadecenoyl cardiolipin molecules (50:35:15 ratio) following the protocol of Parey et al. (18). The model was solvated with 27,911 water molecules, 131 Na cations, and 50 Cl anions (100 mM plus neutralizing cations) (Fig. 1*B*). All amino acids were modeled in their usual protonation states, with histidine protonation states determined by inspection. Cardiolipin was modeled in its –2 protonation state.

**Fig. 2.**
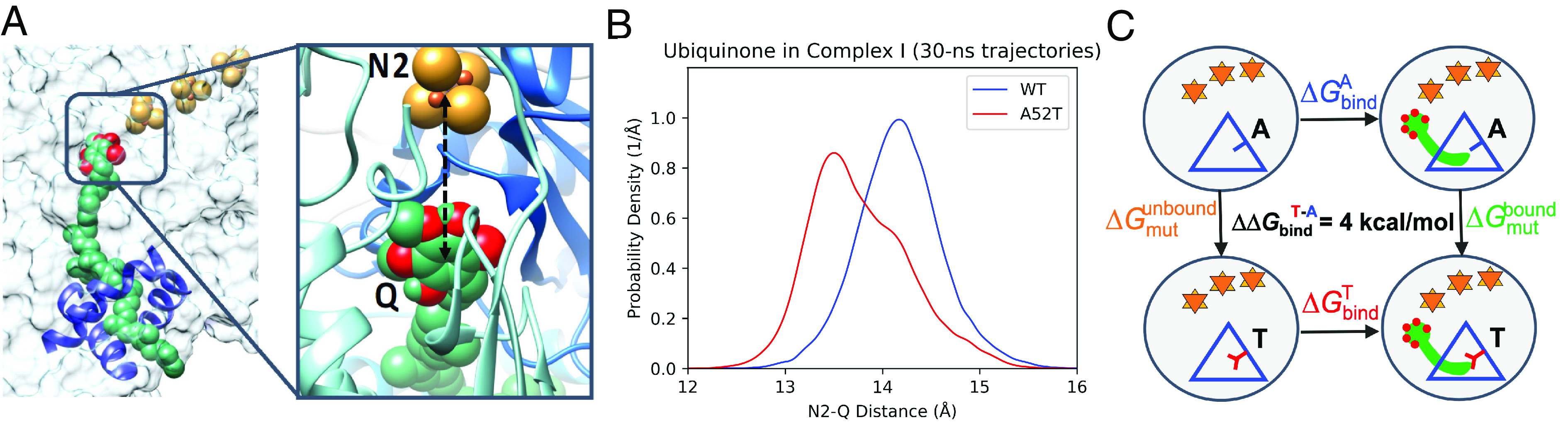
Simulations show that the mutant binds CoQ10 less strongly by ~4 kcal/mol. (*A*) The distance between the center of the CoQ10 headgroup and the center of N2 (black dashed arrow) monitored throughout the molecular dynamics simulation showing (*B*) that shorter distances are accessible to CoQ10 in the mutated pocket (red curve), facilitating a higher rate of electron transfer. (*C*) Thermodynamic cycle for the FEP simulations to compute the relative binding affinity of CoQ10 in WT and A52T.

These simulations show that the mutation neither distorts the protein nor significantly alters the relationship between CoQ10 and the binding channel (Fig. 2*A*). The series of Fe/S clusters remain aligned and equidistant at about 11 Å, to allow for the electron hopping toward CoQ10. However, the position of CoQ10 with respect to the nearest Fe/S cluster (N2) in the m.3460G>A mutant becomes slightly closer (Fig. 2*B*). Specifically, in the WT, the distance between the center of N2 and the center of the CoQ10 headgroup peaks at 14.1 Å, whereas in m.3460G>A, the distance distribution is bimodal with one peak still at 14.1 Å, and another, more prominent peak at 13.5 Å. The bimodal character describes the dynamic behavior in which the CoQ10 substrate rattles in the binding pocket, and frequently visits conformations where it is more proximal to the N2 cluster than can be achieved in the WT. Since the tunneling rate is strongly affected by the tunneling barrier width, the tightened contact between N2 and CoQ10 is expected to enhance electron transfer by narrowing the tunneling barrier. Based on Marcus theory, the tunneling rate in the mutant is estimated to be ca. a factor of 3 greater than in WT (*SI Appendix*). The conformations placing the head group at 13.5 Å from N2 will dominate the electron transfer in the mutant. Electron transfer can go both ways: In its oxidized form, CoQ10 accepts electrons and protons upon reduction, constituting the normal function of the complex. In the reduced form, CoQ10H_2_ may transfer electrons backward up the series of Fe/S clusters, resulting in the production of ROS and failure to move the electrons to complex III. We do not explicitly simulate the electron transfer in this work, as this effect appears secondary in the mechanism of mutation-induced change of the ND1 function (vide infra).

In order to estimate the relative binding free energies of CoQ10 to the WT and the mutant protein, we performed free energy perturbation (FEP; ref. 19) simulations (*SI Appendix*), in which the mutation was alchemically introduced in the channel. The mutation is analyzed with the CoQ10 unbound and bound. By completing the thermodynamic cycle shown in Fig. 2*C*, the otherwise intractable difference in the binding free energies of the two protein variants can be calculated as follows:

$$\begin{array}{c} \Delta \Delta G_{\mathrm{bind}}=\Delta G_{\mathrm{bind}}^{A}-\Delta G_{\mathrm{bind}}^{T}=\Delta G_{\mathrm{mut}}^{\mathrm{unbound}}-\Delta G_{\mathrm{mut}}^{\mathrm{bound}}. \end{array}$$

This shows that the m.3460G>A mutant appears to bind CoQ10 ca. 4 kcal/mol less strongly than the native variant of ND1, suggesting that the binding of the oxidized CoQ10 to the mutant would be relatively disfavored. The difference in the binding affinity does not appear to result from any specific gained interaction but from a collection of subtle structural changes. One likely explanation is the better solvation of threonine compared with alanine, when the pocket is void of CoQ10. The natural question is whether or not the mutant would bind CoQ10 at all. Likely it will because the affinity of the WT is more than likely greater than 4 kcal/mol, and thus, the mutant also has some affinity. However, this result is overshadowed by the more important effect of the kinetics.

**Fig. 3.**
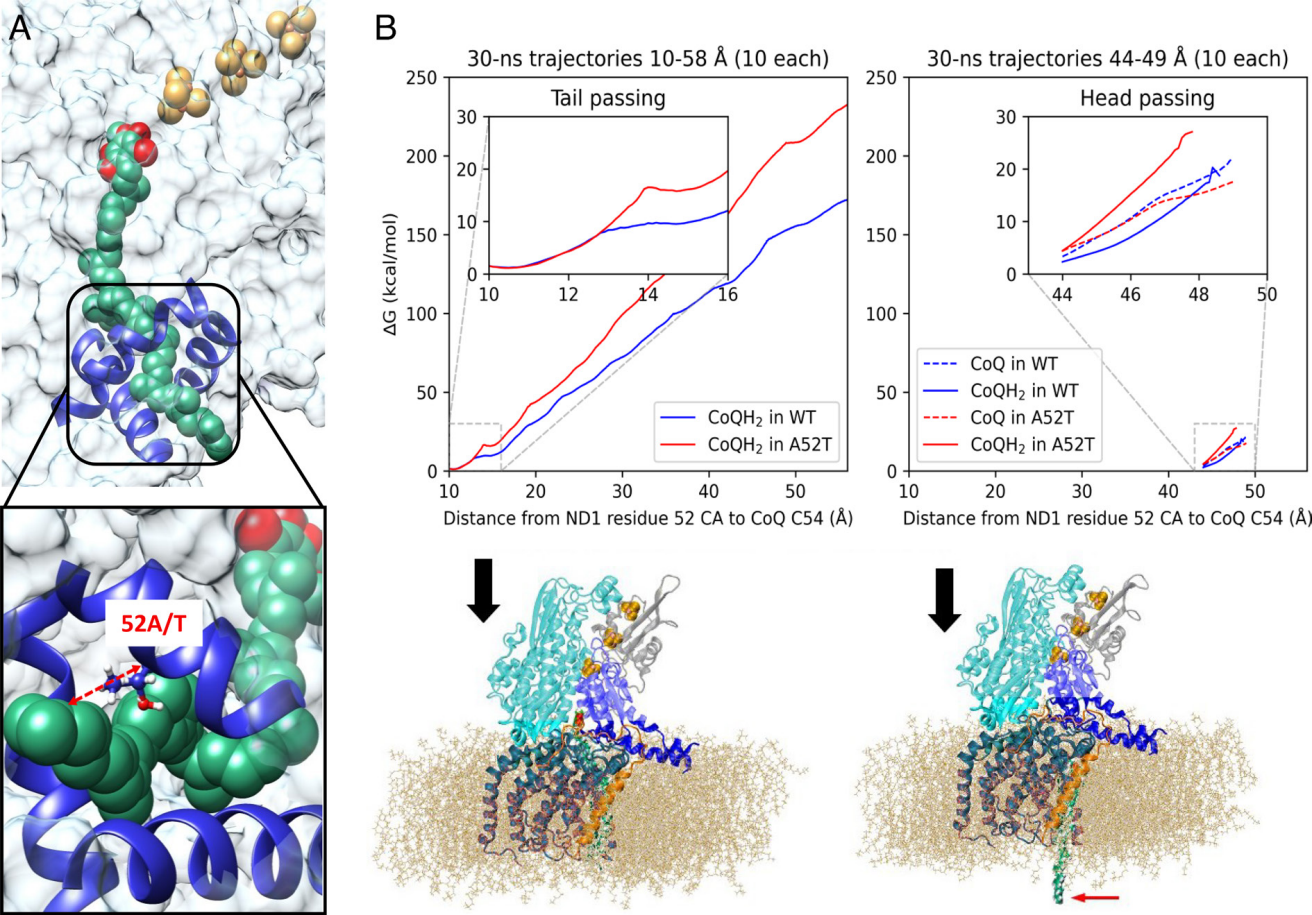
CoQ10 extraction from its ND1 binding pocket. The ND1 protein forms part of the 38-Å-long CoQ10 binding tunnel of respiratory complex I. (*A*) CoQ10 (shown in green) is fully inserted, with the electron receiving end (reducible oxygens shown in red) in close proximity to the terminal Fe-S complex in the Fe-S cluster chain (shown as yellow/orange group). The mutation site is labeled in the *Inset*. The pulling reaction coordinate is shown as the red dashed arrow in the inset (52A/T). (*B*) The reduced CoQ10H_2_ has been “pulled” out by the tail (red arrow at lower right). Unbound CoQ10H_2_ diffuses within the membrane bilayer to complex 3 where it is oxidized. The full extraction simulation over 48 Å is done over the course of 30 ns (*Left*). The inset corresponds to the tail of CoQ10 (15 Å) passing the mutation site. (*Right*) The head of CoQ10 (15 Å) passing the mutation site, performed over the course of 30 ns, showing a more pronounced hindrance caused by the mutation. The legends indicate whether the trajectory is WT or A52T mutant and whether CoQ10 is oxidized or reduced (*SI Appendix*).

The mutation may alter the kinetics of binding of oxidized CoQ10, and unbinding of the reduced product, CoQ10H_2_, (ubiquinol). This hypothesis is based on the fact that the mutated residue is larger in volume (Fig. 3*A*) and is located at the entry channel of the ND1 protein, where it can hinder the passing of the oxidized and/or reduced forms of the substrate. Starting from equilibrated, bound structures of CoQ10 and CoQ10H_2_ with ND1 and its m.3460G>A mutant, we performed steered molecular dynamics simulations (20–22) to assess the difference in the kinetics (Fig. 3*B*). The distance between the α-carbon of residue 52 (52 CA) of ND1 and the final sp^2^-hybridized carbon of the CoQ10 tail (CoQ C54) was the pulling coordinate (Fig. 3*A*). We equilibrated the systems with a harmonic restraint force constant of 2 kcal/mol/Å^2^ centered at 10 Å (corresponding to the bound state with the tip of the CoQ10 tail extruding from the binding channel). We then tested the tugging force at the tail of CoQ10 required to pull it from the pocket. First, the simulations were performed at several different pulling velocities, and the results shown in Fig. 3 correspond to the entire 48 Å pull over a 30 ns period, averaged over 10 trajectories for each system. It took much more work (approximately ~50 kT at room temperature) to pull CoQ10 out of the mutated channel compared with the wild type. This is a very large energy hurdle and is unlikely to be exceeded by the thermal energy of biological systems (*SI Appendix*). The *Insets* show the result of slower and therefore more accurate simulations, of the aliphatic group in the tail of CoQ10, and the larger head of CoQ10 passing the mutation site. These simulations cover 5 Å in 30 ns. We note that these simulations are very expensive and ideally would need to be performed infinitely slowly and averaged over an infinite number of trajectories, which is clearly impossible. However, the trend persists as the pulling speed is gradually reduced by two orders of magnitude, providing confidence to our qualitative findings. In addition, the hindered kinetics makes chemical sense: The small aliphatic group passes with equal ease in WT and A52T, and the same is expected for all the repeating CH_2_ units within CoQ10. However, the passing of the headgroup is significantly more hindered in the mutant, and we attribute the hindrance to the mechanical effect of the larger threonine residue and the less favorable electrostatic interactions with the threonine sidechain.

Hence, the ubiquinol (reduced CoQ10H_2_), in the setting of the mutation is essentially trapped as it would diffuse out of the mutated channel at a rate of about 2 × 10^−9^ slower than from the WT channel. This makes it nearly impossible for the CoQ10H_2_ to leave the channel, and the channel remains effectively blocked. Thus, the electrons cannot be shuttled in the usual manner and will only leave complex I via the more narrowly placed CoQ10 head to the series of Fe/S clusters by moving backward and spilling out, and at an increased rate due to the narrowing of the tunneling barrier, thus producing ROS.

Where would this matter most? Clinically, it is well known that patients with LHON (including the m.3460G>A mutation) may develop sudden and profound loss of vision in both eyes due to loss of axons in the optic nerve (1). Closer consideration shows that this begins with the smallest axon fibers that occupy the papillomacular bundle in the inferior temporal aspect of the optic nerve head. This makes sense insofar as the metabolic needs are very high in neurons, especially those with long axons (23). Each axon potential results in depolarization that requires the Na^+^/K^+^ ATPase pumps to restore their polarization at a high cost of ATP. Indeed, the brain, weighing about 2% of the total body in humans, consumes about 20% of the oxygen and calories of the body. But biology has provided a partial solution through myelinization of axons which limits the area of transmembrane current flux to the nodes of Ranvier, reducing the metabolic needs of axons by about three orders of magnitude. However, RGC axons in the eye, before becoming the optic nerve, traverse several millimeters across the surface of the retina, where they must remain transparent to allow light to penetrate to the underlying rods and cones. Myelin is opaque to light so myelination begins posterior to the lamina cribrosa of the optic nerve head. This unmyelinated segment makes the RGC axons particularly vulnerable to energy impairments. This problem is made worse in the smallest fibers that suffer from adverse surface area to volume ratios, the former reflecting the metabolic cost of repolarization and the latter the number of mitochondria available (23). So, with LHON, these small fibers are impaired and die first (24).

There is a growing consensus that in LHON, the cause of RGC death comes from an excess of ROS production, not lower bioenergetics. ATP depletion would impair axon function long before leading to a wave of apoptosis as is seen clinically in LHON (4). Furthermore, ATP depletion is relatively minor compared with increased ROS production as noted in cybrid and animal models of LHON (5, 7, 8).

## Conclusions

We used molecular dynamics and FEP simulations to elucidate the mechanistic impact of the m.3460G>A mutation on the function of the ND1 protein, leading to Leber’s hereditary optic neuropathy. These simulations show how, in the case of the m.3460G>A mutation, the alanine to threonine substitution at position 52 leads to the extended CH_3_ and an additional OH in the path of the CoQ10. This creates a kinetic hindrance for CoQ10 to pass through the binding channel. The thermal energy required to move the CoQ10 out of the channel is estimated about 50 kT (*SI Appendix*), far too much to allow diffusion to occur at appreciable rates. Additionally, we find that the CoQ10 bound to the mutant ND1 has dynamical access to conformations where its head group is placed ~0.6 Å closer to the nearest FeS cluster, N2, and such conformations are furthermore predominant for the mutant while inaccessible for the WT. The shorter distance indicates a narrower tunneling barrier, which will exponentially increase the electron tunneling probability between N2 and CoQ10. Since CoQ10 is blocked in the channel, this will effectively prevent the reduced form of CoQ10 from leaving the binding channel and thus bring up the probability of electron transfer in the reverse the direction: from the reduced CoQ10 back to N2.

A biochemical analysis of transmitochondrial cybrids and lymphocytes harboring the m.3460G>A mutation showed that while mitochondrial maximal respiration rate was reduced by ~25%, the underlying proportion initiated specifically in complex 1 dysfunction was a ~79% reduction, with none in complex 2 (3), consistent with much-reduced electron transfer via CoQ10. A more recent analysis of cybrids carrying the m.3460G>A mutation showed defects in the assembly and activity of complex 1 including respiratory deficiency, reduced ATP production, and increased ROS and apoptosis (5). Earlier biochemical studies of the m.3460G>A mutation, including kinetic analysis using ubiquinone analogs, indicated a potential inability of complex 1 to interact with CoQ10 (25, 26).

Studies of the structure and kinetics of the electron transfer chain performed with X-ray diffraction and cryo-EM have confirmed that the ND1 channel is already a very tight fit for CoQ10 (27), as well as indicating two stable binding positions for CoQ10 within the tunnel, with perhaps only one close enough to N2 for effective e-transfer (28). Further, refined investigations with cryo-EM and X-ray crystallography performed on the mutant complex with CoQ10 will possibly provide supporting evidence of the distance of the CoQ10 head group to the N2 FeS cluster. Failed electron transfer will lead to electron spillage and ROS production at the proximal end of the FeS cluster series. ROS are a known cause of the blindness from Leber’s hereditary optic neuropathy, and this work thus links the molecular origin of ROS overproduction to the disease process.

## Methods

Simulations were performed in NAMD (29) with the CHARMM36 force field from July 2020 (30–36). Parameters for 4Fe-4S clusters were taken from ref. 37. Parameters for CoQ10 were taken from ref. 38. Water was modeled as TIP3P (39). All bonds to hydrogen were kept fixed to allow for a 2 fs timestep. The system with oxidized CoQ10 was minimized in four stages of 5,000 steps each. Membrane molecules were minimized first to correct pathological initial geometries. Water and ions were added for the second minimization, and all amino acids were added for the third. Finally, all restraints were removed, releasing 4Fe-4S and CoQ10. All further simulations were performed at 310 K and 1.01325 bar using stochastic velocity rescaling (10) and Nosé–Hoover Langevin piston pressure control (11, 12). The minimized system was equilibrated for 2 ns, after which it was used to create three additional systems: WT with reduced CoQ10 and A52T with both oxidized and reduced CoQ10. These four systems were equilibrated for an additional 10 ns. Two more systems without CoQ10, both WT and A52T versions, were constructed from these simulations and equilibrated for an additional 10 ns.

To analyze the effect of the A52T mutation, we used steered molecular dynamics, wherein a time-dependent external force is applied to investigate CoQ10 unbinding (20), and Jarzynski’s identity (21, 22). We chose the distance between the alpha-carbon of residue 52 of ND1 and the final sp2-hybridized carbon of the CoQ10 tail as the pulling coordinate. We equilibrated the systems with a harmonic restraint force constant of 2 kcal/mol/Å^2^ centered at 10 Å (corresponding to the bound state with the tip of the CoQ10 tail extruding from the binding channel) for 10 ns each. We then began extracting structures, one every 100 ps, from which we started pulling trajectories. We ran trajectories at three different constant pulling velocities for each system. The fastest speed moved the harmonic restraint to 58 Å over 3 ns, the intermediate speed moved it to 58 Å over 30 ns, and the slowest speed moved it to 15 Å over 30 ns. We ran additional trajectories equilibrated at 44 Å and pulled to 49 Å over 30 ns. The values for the harmonic restraint center, the measured coordinate, and the accumulated work were output every 500 fs. We used 0.1 Å bins in the analysis.

We also performed binding free energy calculations using FEP (19), where the perturbation corresponded to the mutation, with the goal of assessing the free energy differences in the binding affinity of CoQ10 between the two variants. We constructed dual-topology structures from equilibrated A52T systems with reduced CoQ10, with oxidized CoQ10, and without CoQ10. Each topology included the sidechains of both alanine and threonine for residue 52 of ND1. We split each FEP simulation into 10 windows, using the values of perturbation parameter l of 0, 0.03125, 0.0625, 0.125, 0.25, 0.5, 0.75, 0.875, 0.9375, 0.96875, and 1. For each window, we performed simulations for both forward and reverse alchemical transformations, with 500 ps of equilibration and 2 ns of data acquisition every 500 fs. The free energy change for each window was calculated using Bennett’s acceptance ratio (40). The transformed sidechains were frozen during the FEP calculations, so probability density corrections were calculated using the k-Nearest Neighbor approach (41). For each of the six endpoints, 2.5 ns of additional simulations were performed with the distance from the respective frozen structure measured every 500 fs. The fourth nearest neighbor was used for final corrections. Visualization was done using UCSF Chimera (42) and Visual Molecular Dynamics, VMD (43).

## Supplementary Material

Appendix 01 (PDF)

## Data Availability

All data, code, and materials used in the analysis are available to any researcher for purposes of reproducing or extending the analysis and are deposited in Zenodo (44).
