# Supplementary material for: Coenzyme Q10 trapping in mitochondrial complex I underlies Leber’s hereditary optic neuropathy
Source: Proc Natl Acad Sci U S A. 2023 Sep 21;120(39):e2304884120. doi: 10.1073/pnas.2304884120 (PMC10523484; doi:10.1073/pnas.2304884120)
Supplement: Supplementary file 1 — Appendix 01 (PDF) [file pnas.2304884120.sapp.pdf]

Supporting Information Appendix

Limitations of the computational methodology:

We calculated the equilibrium probabilities of observing various states of the CoQ10 system, where a "state" is defined by the value of a specific interatomic distance. In general, the least likely state along a series of states should dominantly affect the distribution of times (and therefore the average time) it takes for a system at equilibrium to move from the first state to the last state in the series. In our system, this corresponds to the rate at which CoQ10 exits the binding channel of the enzyme. Following conventions in the field of chemistry, we call the least likely state the transition state and quantify the probability difference as the free energy barrier.

The results of this paper are based on the Jarzynski Identity (*1*). This identity states that equilibrium (time-independent) properties can be calculated from non-equilibrium (finite time) trajectories. The practical difficulty in applying this identity is that it requires the population average of a specific quantity over the trajectories. The distribution of the population of that quantity is so skewed that, without extensive sampling to sufficiently observe rare events, the sample average is not quantitatively correct.

According to the Jarzynski Identity, the timescale of the simulations does not affect the final result or the calculated free energy barrier at the transition state. However, the Jarzynski Identity only applies to the population average. In practice, the timescale of the simulations affects the convergence of the sample average to the population average. Slower simulations require fewer trajectories for the quantity of interest to converge. The distribution resulting from faster trajectories is more skewed, with most trajectories requiring extra energy and a few outliers that require much less. With high probability, the sample average describes typical trajectories at a given timescale. The extra energy required by typical trajectories can then be understood as timescale-dependent "friction".

This can be seen in our results, as values from our slowest trajectories show the least friction and presumably give the best approximations to the true energy barriers, although we doubt that they are sufficiently converged for the values to be absolutely precise. However, we do believe that all the results are qualitatively accurate when comparing wild-type and mutant enzymes. We could rephrase our method as averaging the work performed during a process in a way that converges to the free energy difference in the limit of infinite trajectories but drawing qualitative conclusions as approximations before we reach that limit.

In Figure 3b, the ΔG axis shows the amount of work needed to overcome friction for CoQ10H_2 egress within the specified time period and should not be confused as showing the relative free energy between the two configurations, the former being a quantity that is addressed in Figure 2. The difference in the energy required for egress between the WT and A52T-mutant proteins is extremely high (~50 kT), suggesting that at the speed of this simulation, the egress of CoQ10H_2 from the A52T mutant is far less likely to occur than its egress from the WT. However, within the modelled time scale, the barrier for retracting CoQ10H_2 in WT is insurmountable, meaning that egress over a period of 3 or 30 ns is non-physiological. Unfortunately, what happens at lower speeds of egress cannot be simulated computationally given the time required from available resources.

The biophysics of this process requires a sufficiently rapid turnover of CoQ10 so as to allow delivery of electrons to Complex III and to prevent ROS-producing spillage. The biological timescale of CoQ10 mobility in Complex I is not known but considering the energetics encountered in this simulation, it is much slower that the 30 ns timescales used in these computations. Thus, the data in Fig 2b are considered qualitative, not quantitative. We can confidently say that there is much more friction in the A52T channel than in the WT channel, even if we cannot precisely quantify that difference. But the qualitative observation is sufficient to show that egress of $CoQ10H_2$ from the mutant channel is a much slower process than egress from the WT channel, a situation that leads to more electron spillage and ROS production, thus increasing the risk of blindness.

Our results also include other approximations, including classical force-fields, a truncated enzyme model, and periodic boundary conditions. These approximations lead to inaccuracies but in ways identical for the two systems that we compared, and thus they do not override the qualitative conclusions.

Marcus Theory of Electron Tunneling Kinetics:

Since the edge-to-edge distance between the donor (CoQ10) and acceptor (N2) is greater than 10 Å, we can consider the electronic states of each to be weakly coupled. Further, electron transfer timescales relative to nuclear motion follow the Frank-Condon principle, we can use the following Marcus Theory expression for the rate constant of electron tunneling (*2,3*):

$$k_{et} = \frac{1}{h}\left(\frac{\pi^3}{RT\Delta E_R}\right)^{\frac{1}{2}}(H_{et}^0)^2 e^{-\beta L} e^{-\Delta^{\ddagger}G/(RT)}$$

Here, H_{et}^0 is the electronic coupling matrix element at 0 distance between donor and acceptor, ΔE_R is the energy required to arrange the donor and acceptor into a conformation favorable for charge transfer, and $\Delta^{\ddagger}G$ is the free energy required to activate charge transfer, and is dependent on the reorganization energy ΔE_R. The parameter β depends on the environment of the electron transfer, and for electron transfer in vacuum, takes on a value of ca. 30 nm^{-1}. Since the electron is not tunneling through a molecule from CoQ10 to N2, we can use this value for β in vacuum. Lastly, the parameter L is the edge-to-edge donor-acceptor distance, which can be determined by the peaks in Figure 2b.

Due to the wild-type and mutant structures of ND1 not affecting the donor and acceptor molecular structure, we can treat the electronic coupling H_{et}^0 to be the same between both wild-type and mutant ND1. Assuming that there is no further rearrangement cost of CoQ10 for ND1 wild type and mutant, we can estimate that the ratio of k_{et} for wild-type (WT) and mutant (MUT) using the dominant peak only is:

$$\frac{k_{et}(WT)}{k_{et}(MT)} \approx 0.16$$

More accurately, if we consider a mean donor-acceptor distance for the mutant that is a weighted sum of both 13.5 Å and 14 Å peaks in Figure 2b, the ratio is:

$$\frac{k_{et}(WT)}{k_{et}(MUT)} \approx 0.32$$

This provides a strong implication that the reaction rate of electron tunneling, with simple assumptions, is higher for the mutant ND1, and given that the mutant ND1 causes CoQ10 to get kinetically trapped in the binding pocket, the probability of back-tunneling of the electron through the Fe-S cluster increases markedly.

FEP data

**ubiquinol**	ΔG_0 (kcal/mol)	ΔG_1 (kcal/mol)
mutant	17.87	-17.87
window1	1.79204	-1.72406
window2	1.83894	-1.80761
window3	4.07474	-3.97149
window4	8.75676	-8.32546
window5	19.2667	-19.3373
window6	0.632057	-0.48262
window7	0.7987	-0.713948
window8	0.576878	-0.569321
window9	0.324491	-0.343547
window10	0.445338	-0.389718
WT	-3.23	3.23
total	17.406644	-16.565074
lambda		
0	0	0.84157
0.03125	1.79204	2.56563
0.0625	3.63098	4.37324
0.125	7.70572	8.34473
0.25	16.46248	16.67019
0.5	35.72918	36.00749
0.75	36.361237	36.49011
0.875	37.159937	37.204058

0.9375	37.736815	37.773379
0.96875	38.061306	38.116926
1	38.506644	38.506644

FIGURE S1 NEAR HERE.

Figure S1. FEP simulations results for the alchemical mutation on the protein in the presence of bound ubiquinol (forward and backward simulations are shown – the close agreement between the two indicates convergence of the result).

**ubiquinone**	ΔG_0 (kcal/mol)	ΔG_1 (kcal/mol)
mutant	16.01	-16.01
window1	1.62679	-1.70242
window2	1.6834	-1.76937
window3	3.77133	-3.73895
window4	8.30379	-8.05508
window5	18.12	-18.4254
window6	0.666215	-0.437323
window7	0.735912	-0.697356
window8	0.468739	-0.563217
window9	0.32482	-0.334724
window10	0.377544	-0.372715
WT	-3.19	3.19
total	16.87854	-16.896555
lambda		
0	0	-0.018015
0.03125	1.62679	1.684405
0.0625	3.31019	3.453775
0.125	7.08152	7.192725
0.25	15.38531	15.247805
0.5	33.50531	33.673205
0.75	34.171525	34.110528

0.875	34.907437	34.807884
0.9375	35.376176	35.371101
0.96875	35.700996	35.705825
1	36.07854	36.07854

FIGURE S2 NEAR HERE.

Figure S2. FEP simulations results for the alchemical mutation on the protein in the presence of bound ubiquinone (forward and backward simulations are shown – the close agreement between the two indicates convergence of the result).

**no bound CoQ10**	ΔG_0 (kcal/mol)	ΔG_1 (kcal/mol)
mutant	13.93	-13.93
window1	1.88399	-1.92151
window2	1.99829	-2.07025
window3	4.30317	-4.1739
window4	9.04364	-9.01448
window5	18.3254	-18.7763
window6	0.769292	-0.571749
window7	0.701562	-0.881355
window8	0.635573	-0.565265
window9	0.386933	-0.310988
window10	0.376999	-0.379543
WT	-3.21	3.21
total	21.284849	-21.52534
lambda		
0	0	-0.240491
0.03125	1.88399	1.681019
0.0625	3.88228	3.751269
0.125	8.18545	7.925169
0.25	17.22909	16.939649

0.5	35.55449	35.715949
0.75	36.323782	36.287698
0.875	37.025344	37.169053
0.9375	37.660917	37.734318
0.96875	38.04785	38.045306
1	38.424849	38.424849

FIGURE S3 NEAR HERE.

Figure S3. FEP simulations results for the alchemical mutation on the protein in the absence of the bound CoQ10 (forward and backward simulations are shown – the close agreement between the two indicates convergence of the result).

SI References

1. C. Jarzynski, Nonequilibrium equality for free energy differences. *Phys. Rev. Lett.* **78**, 2690-2693 (1997).
2. R.A. Marcus, On the Theory of Electron-Transfer Reactions. VI. Unified Treatment for Homogeneous and Electrode Reactions. *J. Chem. Phys.* **43**, 679-701 (1965).
3. R.A. Marcus, N. Sutin, Electron transfers in chemistry and biology. *Biochim. Biophys. Acta-Bioenerg.* **811**, 265-322 (1985).
